# Psoriasis of the Tongue

**Published:** 1877-04

**Authors:** 


### ARTICLE XIII.

### *On Psoriasis of the Tongue.*

The following general conlusions are given in a contribution, by Dr. Nedopil, to Langenbeck's *Archiv*, of Vienna, on psoriasis of the lingual and oral mucous membrane, and on the relation of this affection to carcinoma :

1. Psoriasis of the mucous membrane of the tongue and oral cavity is caused through a chronic inflammatory process which takes place in the proper mucous layer.

2. This chronic irrative process results in a new formation of young indifferent cells.

3. These indifferent cells, which lie near to the epithelial layer of the mucous membrane, are converted into true epithelium ; hence results the formation of patches of abnormal, bulky and rapidly regenerated epithelum.

4. The epithelial layer of the mucous membrane, as in the normal condition of things, remains quite passive, and takes no part in the regeneration of these patches.

5. The deeply-seated indifferent cells of new formation are partly converted into shrivelled, scar like tissue, and partly persist as round cells, which may finally displace the proper tissue of the mucous layer.

6. These indifferent cells, when in the substance of the mucous layer, and before they have attained their ultimate position may acquire an epithelial character, and thus carcinoma is developed.

7. When carcinoma is developed from indifferent cells, they take on the mode of nutrition as it met with at other times in the surface cells.—*Med. and Surg. Reporter.*

